# Regulation of the Soluble Amyloid Precursor Protein α (sAPPα) Levels by Acetylcholinesterase and Brain-Derived Neurotrophic Factor in Lung Cancer Cell Media

**DOI:** 10.3390/ijms231810746

**Published:** 2022-09-15

**Authors:** Hind Al Khashali, Ravel Ray, Kai-Ling Coleman, Sarah Atali, Ben Haddad, Jadziah Wareham, Jeffrey Guthrie, Deborah Heyl, Hedeel Guy Evans

**Affiliations:** Chemistry Department, Eastern Michigan University, Ypsilanti, MI 48197, USA

**Keywords:** soluble amyloid precursor protein α, brain-derived neurotrophic factor, lung cancer, acetylcholinesterase, amyloid beta, PKC, ERK1/2, PI3K, PKA

## Abstract

In comparing two human lung cancer cells, we previously found lower levels of acetylcholinesterase (AChE) and intact amyloid-β40/42 (Aβ), and higher levels of mature brain-derived neurotrophic factor (mBDNF) in the media of H1299 cells as compared to A549 cell media. In this study, we hypothesized that the levels of soluble amyloid precursor protein α (sAPPα) are regulated by AChE and mBDNF in A549 and H1299 cell media. The levels of sAPPα were higher in the media of H1299 cells. Knockdown of AChE led to increased sAPPα and mBDNF levels and correlated with decreased levels of intact Aβ40/42 in A549 cell media. AChE and mBDNF had opposite effects on the levels of Aβ and sAPPα and were found to operate through a mechanism involving α-secretase activity. Treatment with AChE decreased sAPPα levels and simultaneously increased the levels of intact Aβ40/42 suggesting a role of the protein in shifting APP processing away from the non-amyloidogenic pathway and toward the amyloidogenic pathway, whereas treatment with mBDNF led to opposite effects on those levels. We also show that the levels of sAPPα are regulated by protein kinase C (PKC), extracellular signal-regulated kinase (ERK)1/2, phosphoinositide 3 Kinase (PI3K), but not by protein kinase A (PKA).

## 1. Introduction

Excluding small cell carcinoma, non-small cell lung carcinoma (NSCLC) includes squamous cell carcinoma, large cell carcinoma, and adenocarcinoma [1]. Despite extensive advancement in our understanding of the underlying mechanisms of NSCLC tumor progression, the overall rate of survival remains low with poor prognosis for patients with metastatic disease [2,3]

Evidence has been accumulating showing a number of overlapping molecular pathways and links between cancer and neurodegenerative disease mechanisms [4,5,6]. Many studies have shown an inverse correlation between the likelihood of developing a neurodegenerative disorder and cancer, with those suffering from a neurodegenerative disorder reported to have a lowered incidence for most cancers [4,5,6].

Amyloid precursor protein (APP) is a type 1 transmembrane glycoprotein that is widely recognized for its involvement in the pathogenesis and progression of Alzheimer’s disease (AD), and neuronal homeostasis [7,8,9]. While APP and its processing to amyloid beta (Aβ) have been more extensively studied in AD, the protein is also reported to be expressed ubiquitously by neuronal and non-neuronal cells with frequent overexpression in multiple cancers including lung, prostate, colon, breast, glioblastoma, and pancreatic cancer, driving cancer cell proliferation [7,10]. Structurally, APP is composed of an extracellular domain, an Aβ domain, and a cytoplasmic region that mediates interactions with several proteins [7].

**Figure 1:**
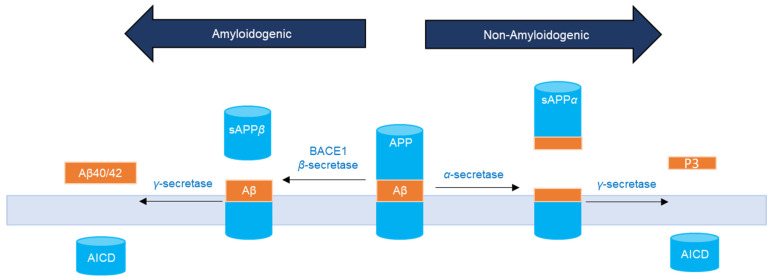
APP processing by two pathways. In the non-amyloidogenic pathway, α-secretase cleaves in the middle of the Aβ region (orange) to release sAPPα. In the amyloidogenic pathway, cleavage of APP by β- and γ-secretase yields Aβ40/42 peptides.

APP can undergo sequential site-specific proteolytic cleavages (Figure 1) via an amyloidogenic or a non-amyloidogenic pathway into biologically active fragments [8,11]. Both pathways are known to be present in virtually all cell types [7,12,13]. Commitment to either pathway appears to be highly regulated by extracellular and intracellular signals [14]. In the amyloidogenic pathway, APP is first cleaved by the membrane-bound aspartyl protease, β-site APP cleaving enzyme I (BACE1, β-secretase), within its extracellular domain and within the NH2 terminus of Aβ to release the 100 kD soluble NH2 terminus ectodomain fragment, sAPPβ [8,12,13,15]. Subsequently, the APP fragmented by BACE1 is cleaved by γ-secretase within the transmembrane domain of APP releasing Aβ40 and Aβ42 and the APP intracellular domain (AICD) [7,8,12,15]. In the non-amyloidogenic pathway, APP is mostly cleaved within the Aβ sequence at the plasma membrane by α-secretase releasing sAPPα, and then by γ-secretase to generate the P3 fragment and AICD, none of which are amyloidogenic [9].

In an attempt to identify regulators of intact Aβ40/42 levels in the media of human A549 (p53 wild-type) and H1299 (p53-null) NSCLC cell lines [16,17], we have previously reported higher intact Aβ levels in the media of A549 than H1299 cells [18]. The difference in these levels was, in part, due to the possible existence of more proteolytic degradation of Aβ40 and Aβ42 in the media of H1299 cells by the matrix metalloproteinase, MMP2 [18].

We also found that the levels of acetylcholinesterase (AChE) were minimal in H1299 cell media as compared to the media of A549 cells [19]. AChE, a member of the serine hydrolase family, is well-known for its classical key role in the catalytic hydrolysis of cholinergic neurotransmitters [20]. Recent studies, however, have attributed non-classical functions to the enzyme as a potential regulator of apoptosis and promising tumor growth suppresser [21] and anticancer therapeutic [22,23]. Lack of AChE expression has been reported in some tumor cells that are not sensitive to apoptosis induction suggesting that decreased AChE levels act to protect the cells against apoptosis [21,22,23].

More recently, we examined the role of mature brain-derived neurotrophic factor (mBDNF) in lung cancer cells as a molecular player implicated in overlapping mechanisms converging at the interface of cancer and neurodegeneration [24]. Our findings showed that the levels of mBDNF are higher in the media of H1299 cells than in A549 cell media [24]. While numerous studies on neurodegenerative disorders have highlighted the role of mBDNF in the survival of neurons and synapses [25,26], emerging roles of neurotrophins in a wide range of cancers, are being increasingly recognized [25,27,28]. Secretion of mBDNF has been shown in tumor cells, increasing cell growth and survival of a range of cancers, including lung [29,30].

Based on our reported observations showing lower levels of intact Aβ40/42 [18] and AChE [19] and higher levels of mBDNF [24] in H1299 cell media, we hypothesized that sAPPα levels are regulated by AChE and mBDNF in the media of A549 and H1299 human NSCLC cell lines and identified signaling pathways involved in this regulation.

## 2. Results

### 2.1. Cell Treatment with the AChE Inhibitor, Neostigmine, Led to Increased sAPPα Levels in A549 Cell Media That Were Comparable to Those Detected in the Media of H1299 cells

**Figure 2:**
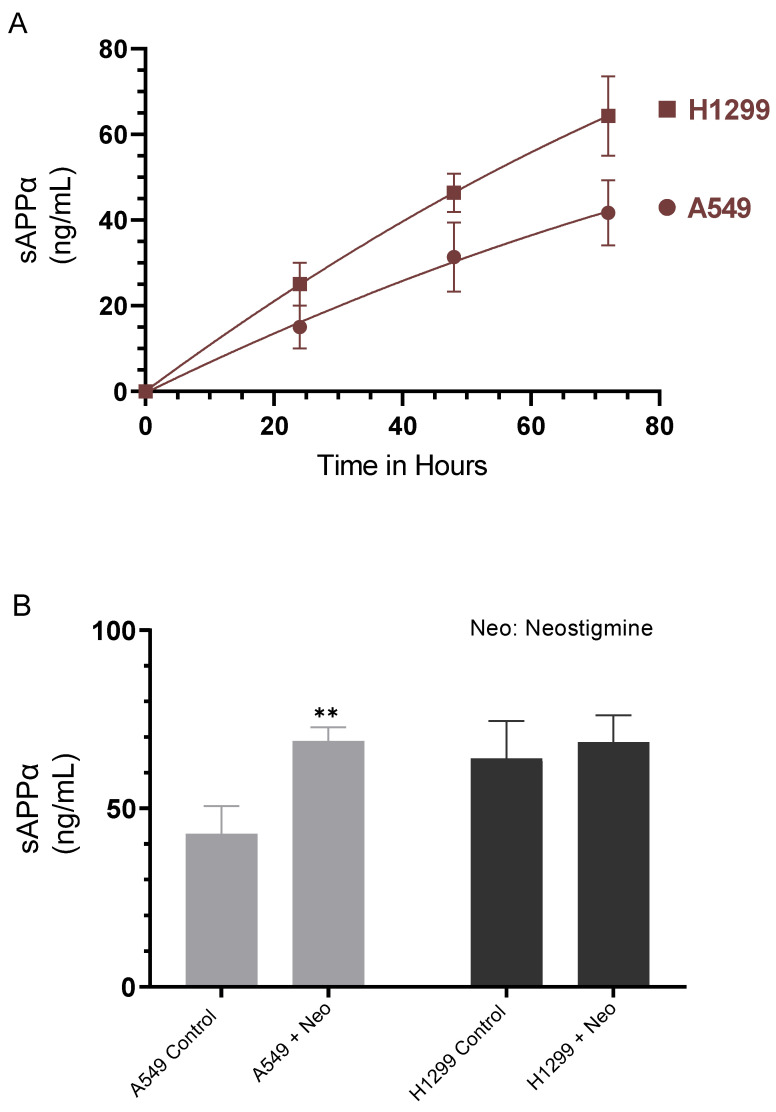
Cell treatment with neostigmine increased sAPPα in A549 cell media to levels comparable to those measured in H1299 cell media. Cells (0.2 × 10^5^) were grown in 10% FBS-supplemented media for 24 h. The following day, the cell monolayers were incubated in serum-free media for the indicated times (**A**) and the levels of sAPPα were measured as described in the Methods section. The levels of sAPPα (**B**) were measured in the media of cells grown as in (**A**) and incubated without or with 50 μM neostigmine (Neo) for 72 h. The same amount of protein (3 µL of 600 µg/mL total protein) of the media was used for the quantitation. Data from five independent assays, each carried out in triplicate, were averaged using the GraphPad 9.4.1 software. The graphs summarize the results expressed as means ± SD (n = 5). Asterisks indicate a statistically significant difference from the corresponding controls, ** *p* < 0.01 of each cell line. Absence of asterisks indicates no significance, Mann–Whitney test.

Several reports have shown that sAPPα, comprising almost the entire extracellular region of APP, has mitogenic effects in fibroblasts, pancreatic, and colon cancers [7,10,13,31,32,33]. We therefore examined the levels of sAPPα in the media of A549 and H1299 cells (Figure 2A). Cells were grown in 10% FBS-supplemented media for 24 h. The following day, the cell monolayers were incubated in serum-free media for the indicated times and the levels of sAPPα were measured as described in the Methods section. The levels of sAPPα were found to be ~1.5-fold higher in the media of H1299 cells as compared to A549 cell media at all the time points examined (Figure 2A).

Among major neurotransmitters, ACh, and stimulation of ACh receptors have been shown to control APP proteolytic processing [34]. Increased brain levels of ACh were found to decrease Aβ in a mouse model of AD [35]. The non-amyloidogenic pathway was shown to be stimulated by nicotinic compounds and activation of nAChRs, including α7nAChR, was found to lead to enhanced APP cleavage to increase sAPPα levels [34,36]. Moreover, using rat brain slices, earlier studies reported that inhibition of AChE resulted in increased APP secretion [37]. 

To examine the possible effects of AChE on sAPPα levels in A549 and H1299 cell media, cells were incubated without or with neostigmine, known to indirectly activate both nicotinic and muscarinic receptors by blocking the active site of AChE, increasing ACh levels and amplifying its effects [38]. Treatment of A549 cells with neostigmine resulted in ~1.6-fold increase in the levels of sAPPα in the media while no difference was observed upon the same treatment of H1299 cells (Figure 2B). The lack of a significant increase in sAPPα levels in H1299 cell media might reflect the relatively minimal levels of AChE in the media of H1299 cells as compared to A549 cell media [19]. 

### 2.2. Knockdown of AChE Led to Increased ACh and mBDNF Levels in A549 Cell Media

Lung cancer cells are known to secrete ACh into the extracellular environment resulting in increased cancerous cell growth in lung tumors [39,40]. By binding to nicotinic and muscarinic receptors on lung cancer cells, ACh acts as an autocrine growth factor accelerating cell proliferation, migration, and invasion [41]. The mitogenic effects of ACh were reported in A549 and H1299 cells, resulting in increased expression of matrix metalloproteinases, and decreased E-cadherin expression in A549 cells [21,41]. The basal ACh concentration secreted by human lung cancer cells was found to be in the 5–50 nM range [41].

While AChE does not appear to initiate apoptosis, several reports have shown that it acts as a tumor suppressor, in part, by the catalytic hydrolysis of Ach [21,42,43,44]. Decreased activity of AChE was reported in lung cancer, likely contributing to increased ACh levels, lung cancer tumor growth rate and aggressiveness, grim prognosis, and reduced survival chance [21,42,43,44]. Increased AChE expression was found during apoptosis in different cell types, and pharmacological inhibition of AChE or blocking its expression by siRNA led to diminished apoptosis [21,45]. AChE may act as a pro-apoptotic gene in NSCLC cells, decreasing cell growth when its expression is upregulated [21,43]. 

**Figure 3:**
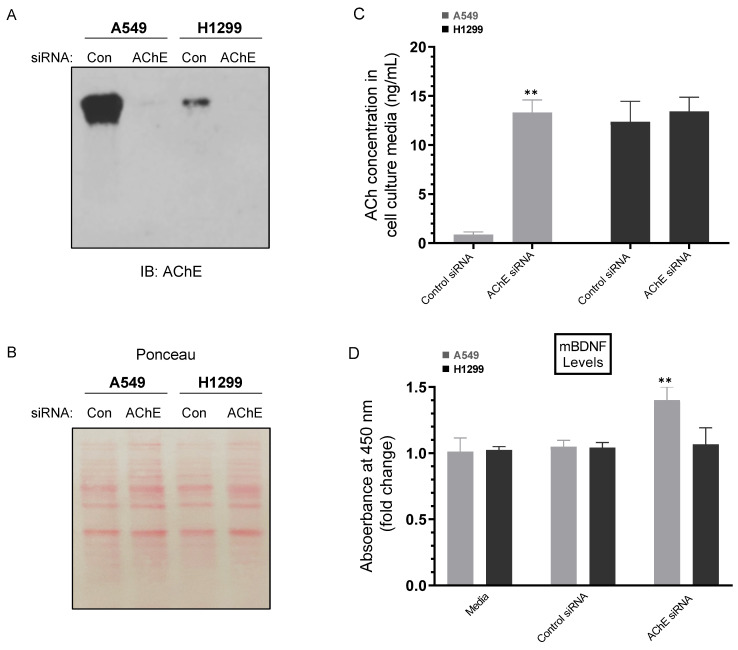
Treatment of cells with AChE siRNA resulted in higher levels of ACh and mBDNF in A549 cell media compared to control siRNA. Cells (0.2 × 10^5^) were grown in 10% FBS-supplemented media for 24 h. The following day, the cell monolayers were incubated in serum-free media for 24 h, then treated for 72 h with the indicated siRNAs as described in the Methods section. The same concentration of total protein (15 µL of 600 µg/mL) of the media (**A**) was used for Western blotting using the indicated antibody (IB: AChE). Total protein (Ponceau staining) (**B**) served as a loading control. The same amount of protein (3 µL of 600 µg/mL total protein) of the media was used to quantitate the levels of ACh (**C**) and mBDNF (**D**) (Methods). Data from five independent assays, each carried out in triplicate, were averaged (**C**,**D**), normalized and expressed as fold change (**D**) relative to cells transfected with control siRNA using the GraphPad 9.4.1 software. The graphs summarize the results expressed as means ± SD (n = 5). Asterisks indicate a statistically significant difference from the corresponding samples transfected with control siRNA, ** *p* < 0.01 of each cell line. Absence of asterisks indicates no significance, Mann–Whitney test.

To determine the concentration of ACh in the media, A549 and H1299 cells were grown in 10% FBS-supplemented media for 24 h. The following day, the cell monolayers were incubated in serum-free media for 24 h, then treated for 72 h with the indicated siRNAs (Figure 3) as described in the Methods section. The levels of ACh were higher in the media of H1299 cells (~12.5 ng/mL) than in A549 cell media (~0.90 ng/mL) upon transfection using control siRNA (Figure 3A–C). Transfection using AChE siRNA led to higher levels of ACh in the media of A549 cells (~13.5 ng/mL) while no significant effects were observed in those levels in H1299 cell media under these conditions (Figure 3C). Since AChE functions to hydrolyze ACh, we expected that treatment with AChE siRNA should result in increased ACh levels, an expectation supported by the higher levels measured in A549 cell media (Figure 3C). The lack of effect on the levels of ACh in the media of H1299 cells is not surprising since the levels of the enzyme are minimal in H1299 cell media relative to those found in the media of A549 cells (Figure 3A,B), a result in accord with our previously published reports [19,46,47].

We also set out to determine the effect of cell treatment with AChE siRNA on the levels of mBDNF (Figure 3D). Previous reports have shown that treatment with the AChE inhibitor, donepezil, was associated with increased serum BDNF levels in AD patients suggesting that AChE inhibitors act to increase BDNF levels leading to neuroprotective effects [48]. In addition, steady AChE inhibition resulted in decreased AChE activity and enhanced BDNF levels in the hippocampus [49]. While no significant change in mBDNF levels were observed in H1299 cell media, there was a ~1.4-fold increase in those levels in the media of A549 cells transfected with AChE siRNA compared to control siRNA transfected cells (Figure 3D). These results suggest that AChE acts to suppress the levels of mBDNF in A549 cell media.

### 2.3. Lower Levels of Aβ40 and Aβ42 Were Observed in A549 Cell Media upon Transfection with AChE siRNA and Correlated with Higher sAPPα Levels

Our results (Figure 2A) show that the levels of sAPPα were ~1.5-fold higher in the media of H1299 cells as compared to A549 cell media. We also found a ~1.6-fold increase in the levels of sAPPα in A549 cell media upon treatment with the AChE inhibitor, neostigmine (Figure 2B). Several reports have shown that in many cell types, increased sAPPα secretion is paralleled by decreased Aβ production [7,8,11,12,15,50]. We therefore examined whether there is a correlation between the levels of sAPPα and Aβ40/42 upon knockdown of AChE. Cells were grown in 10% FBS-supplemented media overnight. The following day, the cell monolayers were incubated in serum-free media for 24 h, then treated for 72 h with the indicated siRNAs as described in the Methods section. The media was then collected and the same amount of protein of each sample was used to quantitate Aβ and sAPPα (Methods).

**Figure 4:**
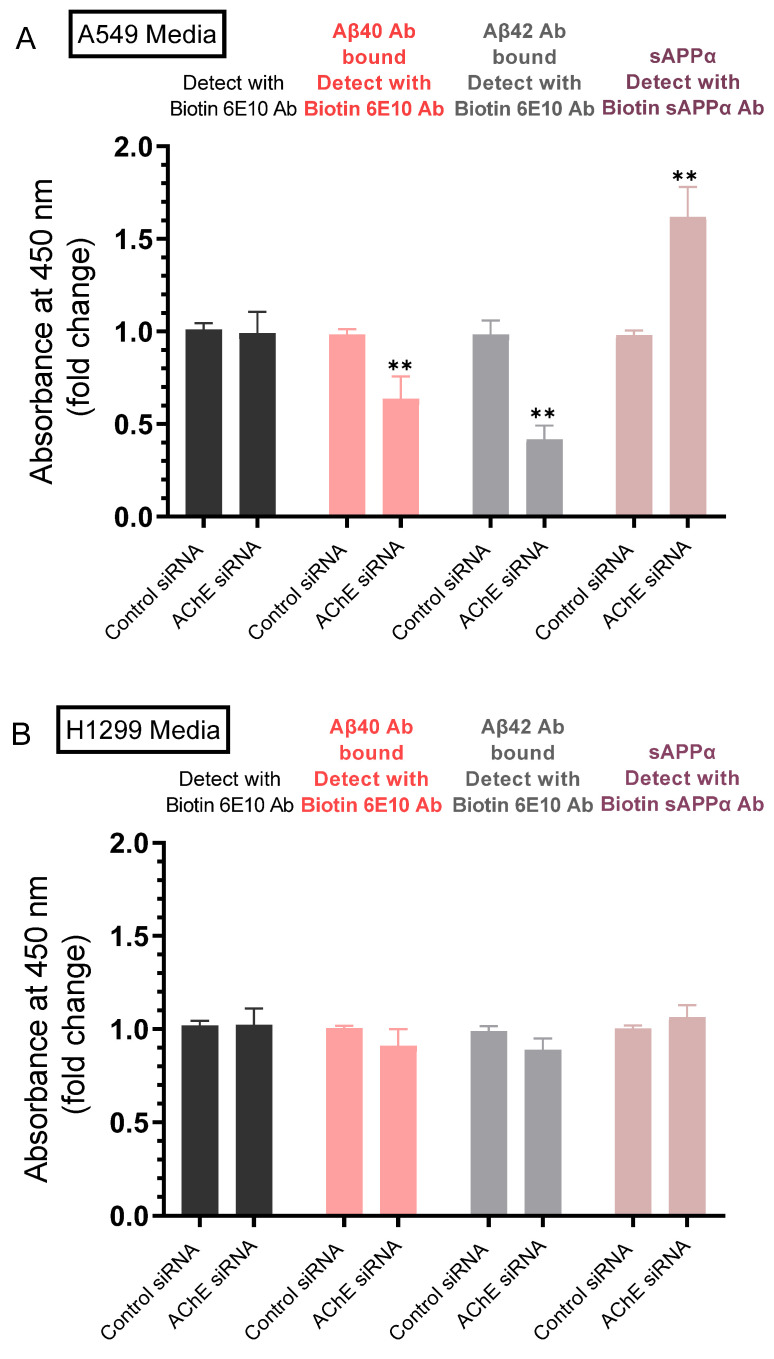
Transfection of A549 cells with AChE siRNA led to lower levels of Aβ40 and Aβ42 which correlated with higher sAPPα levels in the media, effects that were relatively minimal using H1299 cells. Cells (0.2 × 10^5^) were grown in 10% FBS-supplemented media for 24 h. The following day, the cell monolayers were incubated in serum-free media for 24 h, then treated for 72 h with the indicated siRNAs as described in the Methods section. The media from A549 (**A**) and H1299 (**B**) cells was then collected and the same amount of protein (3 µL of 600 µg/mL total protein) of each sample was used to quantitate Aβ and sAPPα (Methods) using the indicated antibodies. Data from five independent assays, each carried out in triplicate, were averaged, normalized, and expressed as fold change relative to cells transfected with control siRNA using the GraphPad 9.4.1 software. The graphs summarize the results expressed as means ± SD (n = 5). Asterisks indicate a statistically significant difference from the corresponding control of each cell line. Absence of asterisks indicates no significance, Mann–Whitney test, ** *p* < 0.01.

Detection using only the biotin 6E10 antibody showed no difference in the signal of A549 and H1299 cells transfected with either control- or AChE-siRNA (Figure 4). The 6E10 antibody is known to recognize an epitope in the first 16 amino acids of the Aβ domain, present in sAPPα, and absent in sAPPβ [51,52,53]. Therefore, the signal detected by this antibody includes that provided by Aβ and sAPPα. Treatment of A549 cells with AChE siRNA resulted in ~1.55-fold and ~2.35-fold decrease in the levels of Aβ40 and Aβ42, respectively and ~1.65-fold increase in the levels of sAPPα (Figure 4A). While the trends were similar with the same treatment of H1299 cells, the effects were not statistically significant (Figure 4B), a finding that might reflect the lower AChE concentration in the media of H1299 cells as compared to that in A549 cell media (Figure 3A,B), a result consistent with our previously published report [19].

### 2.4. AChE and mBDNF Have Opposite Effects on the Levels of Aβ and sAPPα and Operate through a Mechanism Involving α-Secretase Activity

The plasma-membrane anchored protease A Disintegrin And Metalloproteinase, ADAM10, is known as a sheddase that promiscuously cleaves the extracellular domain of a diverse range of substrates from the cell surface [54]. This cleavage generates molecules that trigger signaling cascades involved in growth, cancer pathogenesis, and metastasis [55,56,57]. The main active enzymatic component of α-secretase in primary neurons was shown previously to be ADAM10 [58]. While ADAM17 sheddase activity was comparable in tumors and noncancerous lungs, ADAM10 sheddase activity was reported to be significantly and consistently upregulated in NSCLC cell lines and a potential lung cancer biomarker [59].

Regulation of APP proteolysis has been shown by several reports to be mediated by neurotrophins whereby they can promote non-amyloidogenic APP processing and production of sAPPα while simultaneously precluding the generation and accumulation of toxic Aβ [60,61]. BDNF was shown to decrease APP amyloidogenic processing via a mechanism employing increased α-secretase processing of APP to generate sAPPα [62]. Conversely, APP amyloidogenic cleavage was enhanced by downregulation of cholinergic function [63]. Little is known of the signaling pathways that may regulate cleavage of APP by α-secretase in lung cancer cells.

**Figure 5:**
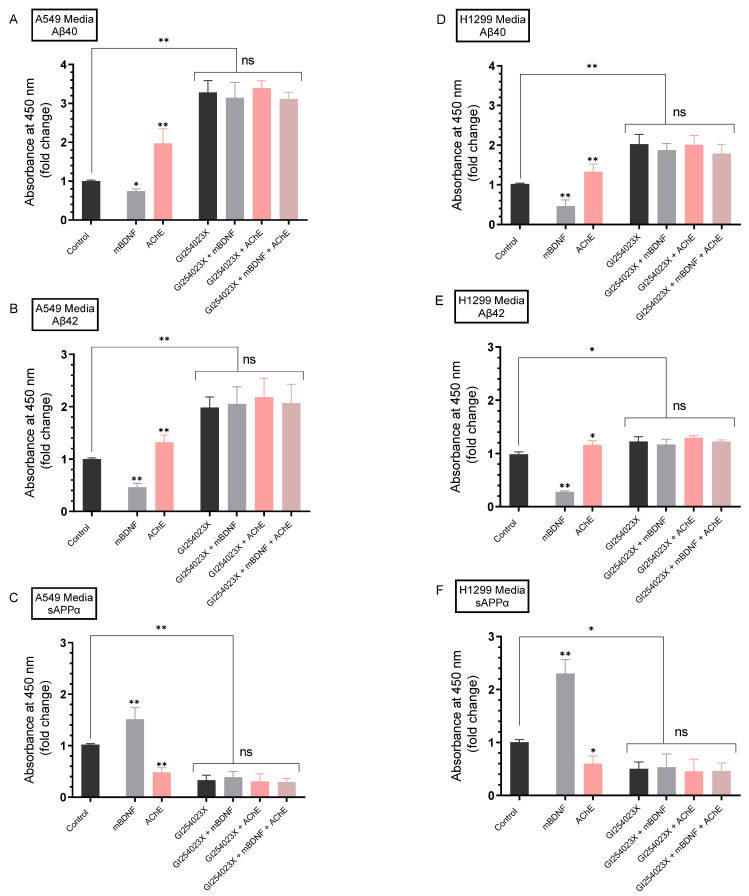
mBDNF decreases the amount of Aβ which correlates with increased sAPPα released from cells in an α-secretase-dependent manner while the converse was observed by AChE treatment. Cells (0.2 × 10^5^) were grown in 10% FBS-supplemented media for 24 h. The following day, the cell monolayers were incubated in serum-free media for 24 h, then treated for 72 h with the ADAM10 inhibitor (GI254023X, 10 μM), mBDNF (5 nM), AChE (60 nM) or in combination. Levels of Aβ40 (**A**,**D**), Aβ42 (**B**,**E**), and sAPPα (**C**,**F**) released into the culture media during the 3-day incubation period were measured as described in the Methods section on the same amount of protein (3 µL of 600 µg/mL total protein) of each sample. Data from five independent assays, each carried out in triplicate, were averaged, normalized, and expressed as fold change relative to untreated cells (control) using the GraphPad 9.4.1 software. The graphs summarize the results expressed as means ± SD (n = 5). Asterisks indicate a statistically significant difference from the corresponding cell line control, * *p* < 0.05, ** *p* < 0.01 of each cell line. Absence of asterisks indicates no significance (ns), Mann–Whitney test.

Our results show that blocking AChE activity with neostigmine (Figure 2B) or knockdown of AChE by siRNA (Figure 4A) led to increased sAPPα levels in the media of A549 cells. Moreover, treatment with AChE siRNA resulted in decreased Aβ40/42 levels in A549 cell media compared to cells transfected with control siRNA (Figure 4A). To examine the possible interplay between ADAM10, mBDNF, and AChE on the levels of Aβ40/42 and sAPPα, A549 and H1299 cells were grown in 10% FBS-supplemented media for 24 h. The following day, the cell monolayers were incubated in serum-free media overnight, then treated for 72 h with the ADAM10 inhibitor (GI254023X), mBDNF, AChE or in combination. Levels of Aβ40, Aβ42, and sAPPα (Figure 5) released into the culture media during the 3-day incubation period were measured as described in the Methods section.

When A549 cells were treated with mBDNF, there was a ~1.32-fold decrease in the levels of Aβ40 (Figure 5A), ~2.15-fold decrease in Aβ42 levels (Figure 5B), and a ~1.50-fold increase in the levels of sAPPα (Figure 5C). The same treatment of H1299 cells with mBDNF resulted in ~2.12-fold decrease in the levels of Aβ40 (Figure 5D), ~3.55-fold decrease in Aβ42 levels (Figure 5E) and a ~2.30-fold increase in the levels of sAPPα (Figure 5F).

Opposite effects to those obtained from mBDNF treatment were observed for both cell lines upon addition of AChE. Treatment of A549 cells with AChE resulted in ~2.05-fold increase in the levels of Aβ40 (Figure 5A), ~1.32-fold increase in the levels of Aβ42 (Figure 5B), and ~2.00-fold decrease in the levels of sAPPα (Figure 5C). Addition of AChE to H1299 cells led to ~1.35-fold and ~1.15-fold increase (Figure 5D,E) in Aβ40 and Aβ42 levels, respectively, while the levels of sAPPα were decreased ~1.55-fold (Figure 5F).

Treatment of A549 cells with GI254023X resulted in a ~3.30-fold increase in Aβ40 levels (Figure 5A), ~1.95-fold increase in Aβ42 levels (Figure 5B), and ~3.05-fold decrease in the levels of sAPPα (Figure 5C). While the effects were relatively smaller using H1299 cells, similar trends were observed upon cell incubation with GI254023X (Figure 5D–F). There was a ~2.05-fold increase in Aβ40 levels (Figure 5D), ~1.20-fold increase in Aβ42 levels (Figure 5E), and ~2.02-fold decrease in the levels of sAPPα (Figure 5F).

For both cell lines and compared to control cells, the levels of Aβ40 and Aβ42 were still higher and the levels of sAPPα were still lower upon co-treatment with mBDNF and GI254023X (Figure 5). These levels were comparable to those using only GI254023X suggesting that mBDNF exerts its effects on Aβ and sAPPα levels through a mechanism that involves α-secretase activity (Figure 5). Moreover, similar results were obtained upon co-treatment of A549 and H1299 cells with AChE + GI254023X or with AChE + GI254023X + mBDNF to those of only GI254023X likely suggesting the involvement of α-secretase activity in this mechanism.

### 2.5. Minimal Effects Were Observed on the Levels of sAPPα in A549 and H1299 Cell Media upon Blocking Protein Kinase A (PKA) Activity

**Figure 6:**
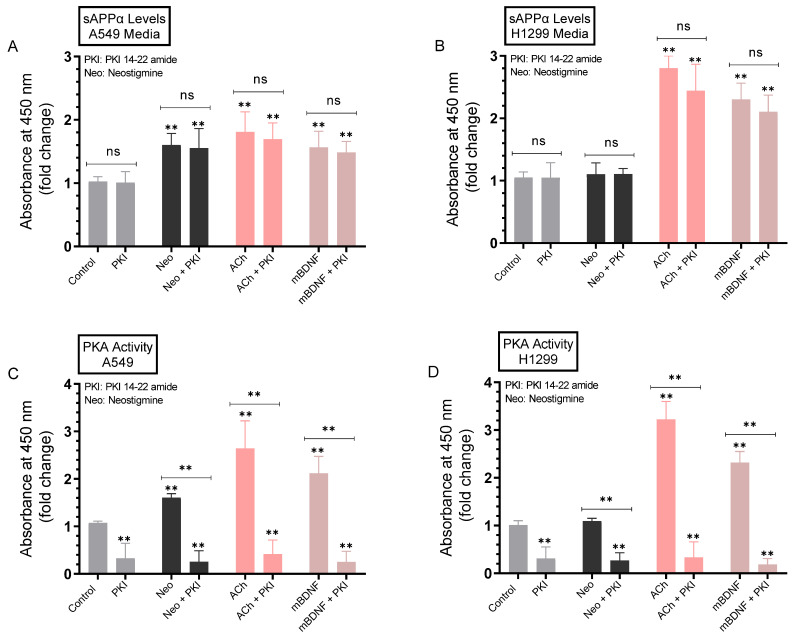
Blocking PKA activity had a minimal effect on the level of sAPPα in A549 and H1299 cell media. Cells (0.2 × 10^5^) were grown in 10% FBS-supplemented media for 24 h. The following day, the cell monolayers were incubated in serum-free media for 24 h, then treated for 72 h with the PKA inhibitor (PKI 14-22 amide, 5 μM), neostigmine (Neo, 50 μM), ACh (100 nM), mBDNF (5 nM), or in combination. Levels of sAPPα released into the culture media of A549 (**A**) and H1299 (**B**) cells during the 3-day incubation period were measured as described in the Methods section on the same amount of protein (3 µL of 600 µg/mL total protein) of each sample. PKA activity was measured in A549 (**C**) and H1299 (**D**) cells as described in the Methods section. Data from five independent assays, each carried out in triplicate, were averaged, normalized, and expressed as fold change relative to untreated cells (control) using the GraphPad 9.4.1 software. The graphs summarize the results expressed as means ± SD (n = 5). Asterisks indicate a statistically significant difference from the corresponding control of each cell line while absence of asterisks indicates no significance (ns). Mann–Whitney test, ** *p* < 0.01.

The levels of sAPPα have been previously shown to be regulated by signaling pathways including protein kinase C (PKC), extracellular signal-regulated kinase (ERK)1/2, and phosphoinositide 3-Kinase (PI3K) [11,64]. To identify potential kinases involved in the regulation of sAPPα levels in the media, A549 and H1299 cells were grown in 10% FBS-supplemented media for 24 h. The following day, the cell monolayers were incubated in serum-free media for 24 h, then treated for 72 h with the PKA inhibitor (PKI 14-22 amide), neostigmine, ACh, mBDNF, or in combination (Figure 6). Levels of sAPPα released into the culture media during the 3-day incubation period along with the PKA activity were measured as described in the Methods section.

No effects were observed on the levels of sAPPα in the media of either A549 or H1299 cells treated with the PKA inhibitor as compared to control untreated cells (Figure 6A,B) despite inhibition of the kinase activity (Figure 6C,D). Treatment of A549 cells with the AChE inhibitor, neostigmine, increased the levels of sAPPα in the media ~1.64-fold, while as expected, no significant increase in those levels were observed in the media of H1299 cells (Figure 6B) that express minimal amounts of AChE [19] (Figure 3A,B). The levels of sAPPα increased in the media of A549 cells treated with ACh (~1.80-fold increase) and mBDNF (~1.50-fold increase) (Figure 6A). Similarly, sAPPα levels increased in the media of H1299 cells treated with either ACh (~2.80-fold increase) or mBDNF (~2.35-fold increase) (Figure 6B). That increase in those levels in H1299 cell media treated with either ACh or mBDNF was ~1.55-fold higher than that observed in the media of A549 cells. Activation of the kinase was observed by ACh and mBDNF in both cell lines (Figure 6C,D), however, while the PKA activity was effectively inhibited when A549 (Figure 6C) or H1299 (Figure 6D) cells were co-treated with the PKA inhibitor, PKI 14-22 amide, no significant effects on the levels of sAPPα were observed in either cell line upon treatment with the inhibitor compared to untreated samples under all conditions tested (Figure 6A,B). These results suggest that PKA is not an important regulator of the levels of sAPPα in the media of either A549 or H1299 cells.

### 2.6. The Levels of sAPPα Decreased in the Media of A549 and H1299 Cells upon Treatment with the PKC Inhibitor, Chelerythrine

**Figure 7:**
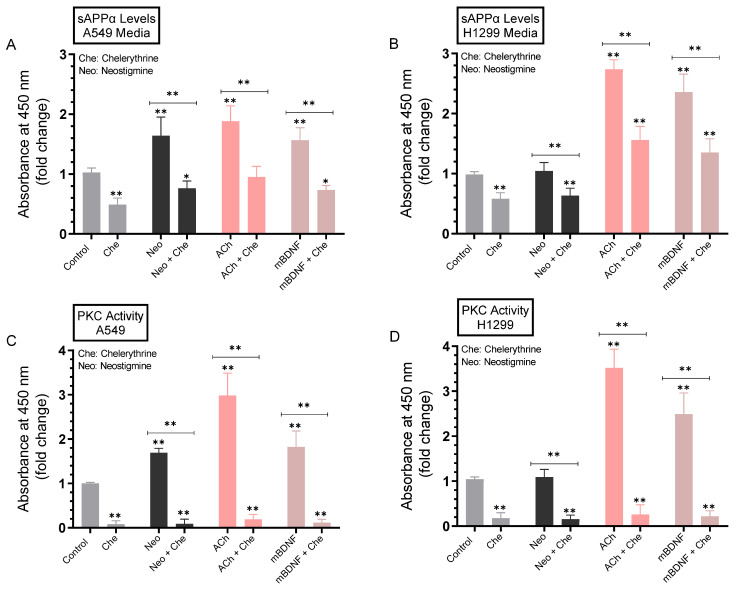
Blocking PKC activity using chelerythrine resulted in decreased sAPPα levels in A549 and H1299 cell media. Cells (0.2 × 10^5^) were grown in 10% FBS-supplemented media for 24 h. The following day, the cell monolayers were incubated in serum-free media for 24 h, then treated for 72 h with the PKC inhibitor (chelerythrine, 7.5 μM), neostigmine (Neo, 50 μM), ACh (100 nM), mBDNF (5 nM), or in combination. Levels of sAPPα released into the culture media of A549 (**A**) and H1299 (**B**) cells during the 3-day incubation period were measured as described in the Methods section on the same amount of protein (3 µL of 600 µg/mL total protein) of each sample. PKC activity was measured in A549 (**C**) and H1299 (**D**) cells as described in the Methods section. Data from five independent assays, each carried out in triplicate, were averaged, normalized, and expressed as fold change relative to untreated cells (control) using the GraphPad 9.4.1 software. The graphs summarize the results expressed as means ± SD (n = 5). Asterisks indicate a statistically significant difference from the corresponding control of each cell line while absence of asterisks indicates no significance. Mann–Whitney test, * *p* < 0.05, ** *p* < 0.01.

To examine the potential contributions of PKC to sAPPα levels, cells were grown in FBS-supplemented media overnight. The cells were then incubated in serum-free media for 24 h, then treated for 72 h with the PKC inhibitor (chelerythrine), neostigmine, ACh, mBDNF, or in combination (Figure 7). Levels of sAPPα released into the culture media of A549 and H1299 cells during the 3-day incubation period and the PKC activity were measured as described in the Methods section. Treatment with chelerythrine resulted in decreased sAPPα levels in the media of A549 cells (~2.0-fold, Figure 7A) and H1299 cells (~1.70-fold, Figure 7B), and effective inactivation of PKC by the inhibitor (Figure 7C,D). Co-treatment of A549 cells with neostigmine and chelerythrine blocked the increased levels of sAPPα found due to cells treated with only neostigmine by ~2.05-fold (Figure 7A). While no significant effects were found when H1299 cells were treated with neostigmine, co-treatment with neostigmine and chelerythrine resulted in ~1.75-fold decrease in sAPPα levels that was comparable to that found for H1299 cells treated with only chelerythrine (Figure 7B). The PKC activity was increased ~1.70-fold in A549 cells treated with neostigmine (Figure 7C) while no effects were found with the same treatment on the kinase activity in H1299 cells (Figure 7D) perhaps reflecting the minimal levels of AChE in H1299 cell media that can be inhibited by neostigmine. Compared to cells treated with either ACh or mBDNF, co-incubation of cells with chelerythrine and either ACh or mBDNF blocked the increased sAPPα levels in A549 cell media by ~2.0-fold (Figure 7A) and by ~1.75-fold in the media of H1299 cells (Figure 7B). These effects paralleled the activity of PKC in both cell lines (Figure 7C,D).

### 2.7. A more Pronounced Decrease in the Levels of sAPPα Was Observed in A549 and H1299 Cell Media When the Activities of ERK1/2 and PKC Were Blocked Using a Combination of PD98059 and Chelerythrine

**Figure 8:**
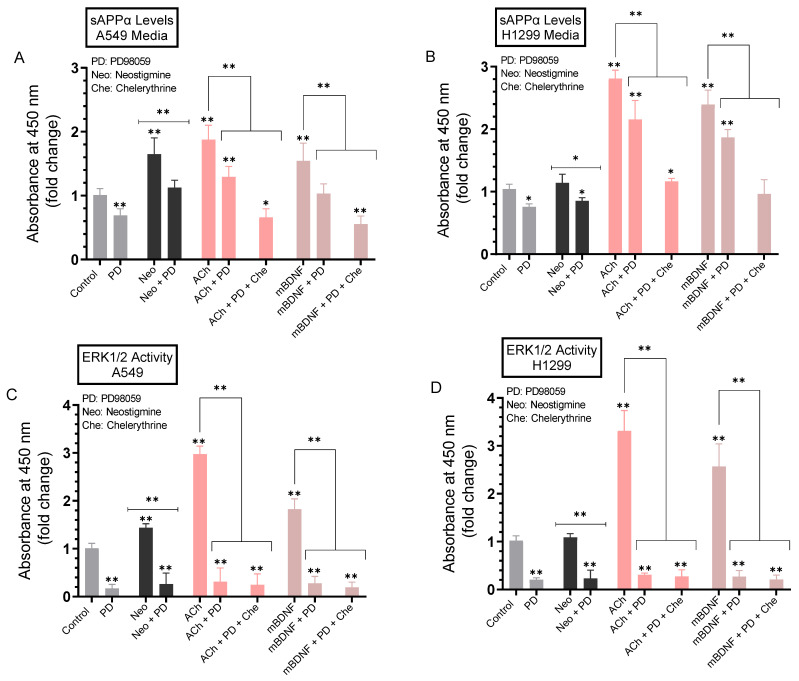
Blocking ERK1/2 and PKC activities using PD98059 and chelerythrine resulted in a larger decrease in sAPPα levels in A549 and H1299 cell media. Cells (0.2 × 10^5^) were grown in 10% FBS-supplemented media for 24 h. The following day, the cell monolayers were incubated in serum-free media for 24 h, then treated for 72 h with the ERK1/2 inhibitor (PD98059, 50 μM) PKC inhibitor (chelerythrine, 7.5 μM), neostigmine (Neo, 50 μM), ACh (100 nM), mBDNF (5 nM), or in combination. Levels of sAPPα released into the culture media of A549 (**A**) and H1299 (**B**) cells during the 3-day incubation period were measured as described in the Methods section on the same amount of protein (3 µL of 600 µg/mL total protein) of each sample. ERK1/2 activity was measured in A549 (**C**) and H1299 (**D**) cells as described in the Methods section. Data from five independent assays, each carried out in triplicate, were averaged, normalized, and expressed as fold change relative to untreated cells (control) using the GraphPad 9.4.1 software. The graphs summarize the results expressed as means ± SD (n = 5). Asterisks indicate a statistically significant difference from the corresponding control of each cell line while absence of asterisks indicates no significance, Mann–Whitney test. Statistical differences between different groups were analyzed by an ordinary one-way analysis of variance (ANOVA) followed by Tukey’s post-hoc multiple comparison test. * *p* < 0.05, ** *p* < 0.01.

To test the effects of blocking the activity of ERK1/2 on the levels of sAPPα in the media, A549 and H1299 cells were grown in FBS-supplemented media overnight. The following day, the cell monolayers were incubated in serum-free media for 24 h, then treated for 72 h with the ERK1/2 inhibitor (PD98059), PKC inhibitor (chelerythrine), neostigmine, ACh, mBDNF, or in combination (Figure 8). Levels of sAPPα released into the culture media during the 3-day incubation period and ERK1/2 activity were measured as described in the Methods section.

The levels of sAPPα were decreased ~1.45-fold in the media of A549 cells treated with PD98059 (Figure 8A) and ~1.25-fold in H1299 cell media under the same conditions (Figure 8B). Reduction of these sAPPα levels in the media of both cell lines correlated with inhibition of ERK1/2 activity by PD98059 (Figure 8C,D). Similar reductions in sAPPα levels were found in the media of A549 and H1299 cells treated with PD98059 and either neostigmine, ACh, or mBDNF compared to the same treatments without PD98059 (Figure 8A,B). To examine the effects of blocking both PKC and ERK1/2 on the levels of sAPPα, cells were treated with ACh or mBDNF in the absence or presence of a combination of PD98059 and chelerythrine. Compared to cells treated with either ACh or mBDNF, treatment of A549 cells with either ACh or mBDNF and both inhibitors decreased the levels of sAPPα ~2.80-fold as compared to the ~1.45-fold reduction in those levels in the media of cells treated with either ACh or mBDNF and PD98059 (Figure 8A). While the trends were similar, more modest reductions of sAPPα levels were found in the media of H1299 cells (Figure 8B). Relative to cells treated with either ACh or mBDNF, co-treatment of H1299 cells with ACh, chelerythrine, and PD98059 decreased the sAPPα levels in the media ~2.45-fold as compared to the ~1.30-fold decrease upon treatment of cells with ACh and PD98059 (Figure 8B). Similarly, treatment of H1299 cells with mBDNF in the presence of both chelerythrine and PD98059 led to ~2.50-fold decrease in the levels of sAPPα in the media as compared to a reduction of ~1.25-fold in the media of H1299 cells treated with mBDNF and PD98059 (Figure 8B).

### 2.8. The Levels of sAPPα Were Decreased to a Greater Extent in the Media upon Co-Treatment of A549 and H1299 Cells with ACh or mBDNF, the PI3K Inhibitor, LY294002, and Either the ERK1/2 Inhibitor or PKC Inhibitor Compared to Cell Co-Treatment with LY294002 Alone

**Figure 9:**
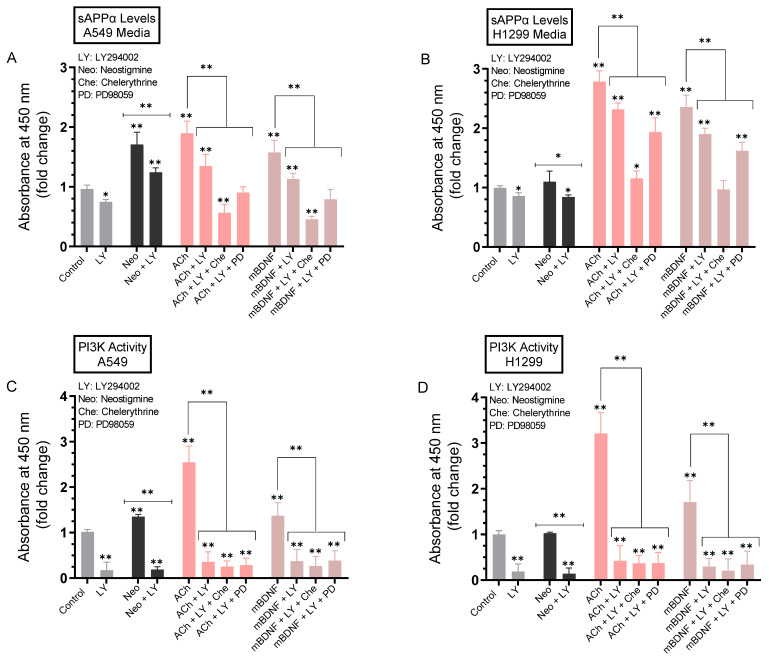
Co-treatment of cells with ACh or mBDNF, the PI3K inhibitor, LY294002, and either the ERK1/2 inhibitor or PKC inhibitor decreased the levels of sAPPα in the media of A549 and H1299 cells to a greater extent than co-treatment with LY294002 alone. Cells (0.2 × 10^5^) were grown in 10% FBS-supplemented media for 24 h. The following day, the cell monolayers were incubated in serum-free media for 24 h, then treated for 72 h with the PI3K inhibitor (LY294002, 14.5 μM) alone or in combination with the ERK1/2 inhibitor (PD98059, 50 μM) or PKC inhibitor (chelerythrine, 7.5 μM), neostigmine (Neo, 50 μM), ACh (100 nM), and mBDNF (5 nM). Levels of sAPPα released into the culture media of A549 (**A**) and H1299 (**B**) cells during the 3-day incubation period were measured as described in the Methods section on the same amount of protein (3 µL of 600 µg/mL total protein) of each sample. PI3K activity was measured in A549 (**C**) and H1299 (**D**) cells as described in the Methods section. Data from five independent assays, each carried out in triplicate, were averaged, normalized, and expressed as fold change relative to untreated cells (control) using the GraphPad 9.4.1 software. The graphs summarize the results expressed as means ± SD (n = 5). Asterisks indicate a statistically significant difference from the corresponding control of each cell line while absence of asterisks indicates no significance, Mann–Whitney test. Statistical differences between different groups were analyzed by an ordinary one-way analysis of variance (ANOVA) followed by Tukey’s post-hoc multiple comparison test. * *p* < 0.05, ** *p* < 0.01.

We next examined the effects of inhibiting PI3K on the levels of sAPPα in the media. Cells were allowed to grow in 10% FBS-supplemented media for 24h. The following day, the cell monolayers were incubated in serum-free media overnight, then treated for 72 h with the PI3K inhibitor (LY294002) alone or in combination with the ERK1/2 inhibitor (PD98059) or PKC inhibitor (chelerythrine), neostigmine, ACh, and mBDNF (Figure 9). Levels of sAPPα released into the culture media of A549 and H1299 cells along with the activity of PI3K during the 3-day incubation period were measured as described in the Methods section.

Incubation of A549 cells with the PI3K inhibitor resulted in ~1.35-fold decrease in the levels of sAPPα while that decrease was ~1.15-fold in the media of H1299 cells treated under the same conditions (Figure 9A,B). Treatment of cells with LY294002 and either neostigmine, ACh, or mBDNF led to a ~1.40-fold decrease in the levels of sAPPα in A549 cell media (Figure 9A) and ~1.20-fold decrease in the media of H1299 cells (Figure 9B) compared to cells not treated with LY294002. Relative to cells treated with either ACh or mBDNF, co-treatment of A549 cells with LY294002 and chelerythrine led to a greater reduction in sAPPα levels in the media, ~3.50-fold decrease, as compared to the ~2.00-fold decrease observed when A549 cells were co-treated with LY294002 and PD98059 (Figure 9A). While the trends were comparable when using H1299 cells, the effects were relatively more modest (Figure 9B). The levels of sAPPα in the media were decreased ~2.45-fold when H1299 cells were treated with ACh or mBDNF, LY294002, and chelerythrine while a smaller decrease in those levels, ~1.45-fold, was found when PD98059 was used instead of chelerythrine under the same conditions (Figure 9B). In both cell lines, there was effective inhibition of the activity of PI3K when cells were treated with LY294002 (Figure 9C,D). Collectively, these results suggest that co-treatment with inhibitors against PI3K and PKC is more effective at regulating sAPPα levels in the media of A549 and H1299 cells than co-treatment with PI3K and ERK1/2 inhibitors.

## 3. Discussion

We have previously reported higher intact Aβ40/42 levels in the media of A549 than H1299 cells that were in part due to more proteolytic degradation of Aβ40 and Aβ42 in the media of H1299 cells by MMP2 [18]. We also found that AChE expression level was minimal in H1299 cells as compared to that in A549 cells [19]. More recently, we reported that the levels of mBDNF were higher in the media of H1299 cells than in A549 cell media [24]. In this study, we hypothesized that the levels of sAPPα are regulated by AChE and mBDNF in A549 and H1299 human NSCLC cell lines.

APP (Figure 1) was reported to regulate global protein synthesis in NSCLC cells among other human dividing cells [65]. APP was found to be required for G0/G1 transitions with an important role in cell cycle entry regulation, and its depletion led to abnormalities in cell size, cell membrane permeabilization, and death [65]. Being a highly pleiotropic protein, APP is involved in a range of functions and processes essential for carcinogenesis that include cell survival [9]. Knockdown of APP led to reduced breast cancer cell motility and growth, in part, by induction of caspase-3-mediated apoptosis [66]. Our results show that the levels of sAPPα were ~1.5-fold higher in the media of H1299 cells as compared to the levels found in A549 cell media (Figure 2A).

AChE inhibitors prevent the hydrolysis of released ACh, thereby increasing the efficiency of cholinergic transmission [67]. Acute treatment of SH-SY5Y neuroblastoma cells with AChE inhibitors was found to result in increased secreted levels of sAPPα into the conditioned media of the cells [68]. These levels were found to be inversely correlated with the levels of AChE inhibition [68]. A549 cells treated with neostigmine, known to block the active site of AChE, increasing ACh levels [38], resulted in ~1.6-fold increase in the levels of sAPPα in the media while no effect was detected upon the same treatment of H1299 cells (Figure 2B). The lack of significant increase in the levels of sAPPα in H1299 cell media is likely due to the minimal levels of AChE in the media of H1299 cells as compared to those detected in A549 cell media [19].

Traditionally regarded as an important neurotransmitter, ACh is known to regulate a range of fundamental processes in the central and peripheral nervous system acting via activation of ACh receptors [67,69]. More recently, several reports have emerged showing that ACh can be widely produced and released by normal and neoplastic non-neuronal cell types highlighting its important role in tumorigenesis with a potential function as a growth factor to stimulate cell proliferation in different types of cancer, including NSCLC [41,70,71]. ACh production and release by colon cancer cells was found to mediate autocrine stimulation of cell proliferation [72]. Similarly in gastric cancer cells, an autocrine loop for ACh was shown suggesting that ACh might function as a growth factor to stimulate cell proliferation [73]. In addition, cholinergic signaling was found to be significantly upregulated in lung tumors as compared to normal lung [21,41,70,74,75]. Lung cancers were found to synthesize and secrete ACh that acts as an autoparacrine growth factor stimulating cell proliferation through nicotinic- or muscarinic-cholinergic pathways [76,77]. Studies have shown choline acetyltransferase (ChAT) upregulation and downregulation of cholinesterases in NSCLC, resulting in increased ACh concentrations [39]. We found that the levels of ACh were higher in H1299 cell media than in the media of A549 cells upon transfection with control siRNA (Figure 3A–C). While no significant effects were observed in the levels of ACh in the media of H1299 cells transfected with AChE siRNA, higher ACh levels were found in the media of A549 cells under these conditions (Figure 3A–C). The lack of effect on the levels of ACh in the media of H1299 cells transfected with AChE siRNA is in accord with our previously published reports showing minimal levels of AChE in the media of H1299 cells relative to those in A549 cell media [19,46,47].

Preclinical and clinical studies have provided evidence for the potential use of AChE inhibitors in the treatment of AD [22,67]. Treatment with donepezil, an AChE inhibitor, was associated with upregulated serum BDNF levels in AD patients mimicking those of healthy control levels, perhaps suggesting that AChE inhibitors exhibit their neuroprotective effects via a mechanism involving increasing BDNF levels [48]. In addition, increased phosphorylation of CREB (cAMP response element binding protein), an important upstream signaling molecule of BDNF, was found upon chronic donepezil treatment of rats [78]. Other studies also found that steady AChE inhibition led to decreased AChE activity and increased levels of BDNF levels in the hippocampus [49]. Moreover, oral administration of the selective AChE inhibitor, huperzine A, increased the levels of BDNF mRNA and protein, decreasing memory deficits and neuronal damage in mice [79]. No significant change in mBDNF levels was observed in H1299 cell media upon knockdown of AChE, however, there was a ~1.4-fold increase in those levels in the media of A549 cells transfected with AChE siRNA (Figure 3D), suggesting that AChE might function to suppress mBDNF levels in A549 cell media.

Several reports have previously demonstrated reciprocal regulation of APP processing into sAPPα and Aβ in that increased secretion of sAPPα is associated with decreased Aβ generation [80,81]. Several major neurotransmitters such as ACh and stimulation of ACh receptors are known to control the proteolytic processing of APP [34]. Nicotinic ACh receptors (nAChRs) or in combination with muscarinic ACh receptors (mAChRs) have also been found to participate in the formation of sAPPα [11]. For example, an increase in APP secretion was observed following mAChR stimulation and was accompanied by both increased generation of the non-amyloidogenic P3 fragment and a concomitant reduction in the release of soluble Aβ [82], findings that led to the hypothesis that activation of M1/M3 mAChR-associated signaling pathways increases α-secretase activity and decreases processing of APP by β-secretase [80]. Using rat brain slices, previous reports have shown that inhibition of AChE led to increased non-amyloidogenic soluble APP secretion [37]. Increased brain ACh was shown to lower Aβ in a mouse model of AD [35]. Other reports found that activation of nAChR may lead to increased APP cleavage to elevate sAPPα and that the non-amyloidogenic pathway can be stimulated by nicotinic compounds [34,36]. The α7nAChR was shown to play a major role in this process [34,36]. Nicotine treatment was found to increase sAPPα release into the media and decrease Aβ levels in the human neuroblastoma cell line, SH-SY5Y, expressing α7nAChR subtypes [36]. Attenuation of β-amyloidosis and stimulation of the non-amyloidogenic pathway by nicotine was found to be mediated by nAChRs with a key major role of α7nAChRs in modulating these processes [36].

Many studies have reported earlier that the activity of AChE was decreased in lung cancer, likely leading to enhanced lung cancer growth [21,42,43,44]. Knockdown of AChE expression by siRNA or blocking the activity of the enzyme using pharmacological inhibitors, decreased apoptosis [21,45]. Treatment of A549 cells with AChE siRNA resulted in decreased levels of Aβ40 and Aβ42, with a concomitant increase in the levels of sAPPα (Figure 4) suggesting that AChE is involved in regulating these levels.

Ectodomain cleavage by ADAMs has been reported as an important key mechanism for modulating receptor functions [15,55]. Sheddases are expressed in many tissues with frequent overexpression in various types of cancer and pre-cancerous lesions [54]. ADAM10 contains a catalytic metalloproteinase domain related to that of MMPs, a disintegrin domain needed in cell adhesion, and a C-terminal cytoplasmic tail with a role in activity regulation [54]. ADAM10 is also known as a zinc MMP that cleaves APP at the α-secretase cleavage site precluding formation of Aβ [8,54]. While ADAM10 and ADAM17 comprise active members of the α-secretase family, it is generally thought that ADAM10 carries out the constitutive cleavage of APP whereas ADAM17 is involved in the regulated processing [11]. Cleavage by α-secretase between Lys16 and Leu17 occurs within the Aβ peptide and can result in the release of sAPPα, the large soluble, nontoxic proteolytic N-terminal ectodomain fragment of non-amyloidogenic APP processing, known for a number of cytoprotective functions both in cell culture and in animal models [7,8,81]. Therefore, APP cleavage by α-secretase not only blocks generation of toxic Aβ peptides but also produces sAPPα with cytoprotective functions [7,8,12,15,50,81]. Inhibition of ADAM10 was found to block the levels of sAPPα and reduce breast cancer cell growth and migration [13]. Co-expression of APP and ADAM10 was found to correlate with unfavorable prognosis and worse survival outcomes for patients with different subtypes of breast cancers [13,83]. In addition, APP cleavage by ADAM10 was shown to have an oncogenic role in breast cancer [13].

Neurotrophins have been reported by several studies to regulate APP proteolysis in that they can promote production of sAPPα via non-amyloidogenic APP processing while simultaneously preventing the generation of Aβ [60,61]. Inhibition of BDNF signaling was shown previously to increase Aβ generation in hippocampal neurons [84]. Using a transgenic AD mouse model and cultured human neural cells, earlier studies demonstrated that BDNF reduced amyloidogenic processing of APP and production of toxic Aβ peptides by a mechanism that involves increased α-secretase processing of APP to liberate sAPPα [62]. On the other hand, amyloidogenic cleavage of APP was increased upon downregulation of cholinergic function [63]. Our results (Figure 5) show that addition of AChE resulted in decreased sAPPα levels with a corresponding increase in the levels of Aβ40 and Aβ42 in the media of both A549 and H1299 cells suggesting a role of the protein in shifting APP processing away from the non-amyloidogenic pathway and toward the amyloidogenic pathway. Conversely, opposite effects on those levels were observed upon mBDNF treatment (Figure 5). To test whether the effects on the levels of sAPPα and Aβ are due, in part, to modulation of α-secretase activity, we used the ADAM10 inhibitor, GI254023X. A549 and H1299 cells were treated without or with GI254023X, mBDNF, AChE, or in combination (Figure 5). Treatment with GI254023X led to increased levels of Aβ40 (Figure 5A,D) and Aβ42 (Figure 5B,E) and corresponded to decreased levels of sAPPα (Figure 5C,F) compared to untreated controls. Our data show that the levels of sAPPα remained reduced and the levels of Aβ40/42 remained higher as compared to control upon co-treatment of cells with mBDNF/GI254023X, with AChE/GI254023X, or with AChE/GI254023X/mBDNF (Figure 5) pointing to the involvement of α-secretase. While the observed effects by AChE are opposite to those of mBDNF, both AChE and mBDNF appear to exert their effects, at least in part, via regulation of α-secretase activity (Figure 5). Taken together, our results point to the activity of α-secretase as part of a mechanism employed by mBDNF and AChE in regulating sAPPα and Aβ levels in the media of A549 and H1299 cells.

**Figure 10:**
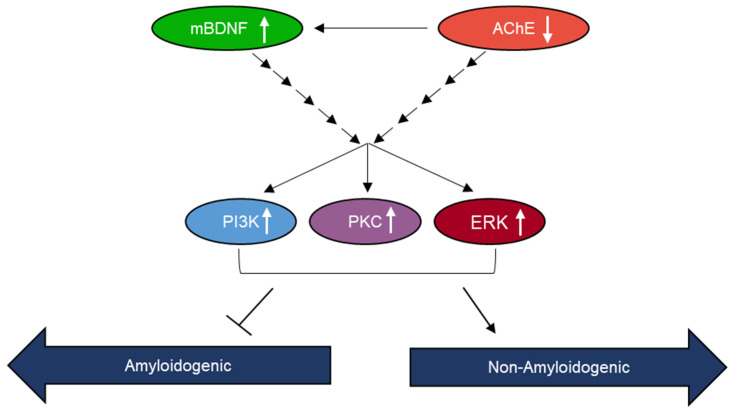
Representation of the main hypothesis and findings of this study.

In an attempt to identify kinases important for regulating the levels of sAPPα, we used inhibitors and found no significant effects on those levels upon blocking PKA activity (Figure 6). Blocking PKC activity, however, resulted in decreased sAPPα levels in A549 and H1299 cell media (Figure 7). Expression of PKC isoforms was found to be higher in NSCLC than in lung epithelial cells [85]. PKC isoform activation has been shown to be linked to proliferation, carcinogenesis and malignant progression of a range of human cancers [85]. PKC signaling was reported previously to be a central mechanism and to play a pivotal role in regulating APP metabolism via both direct and indirect receptor-mediated PKC activation leading to increased sAPPα release and decreased Aβ secretion [11,64]. PKC signaling was shown to be coupled to and activated by muscarinic (M1/M3) ACh receptors stimulating sAPPα release [11,86]. Blocking AChE activity was reported to increase PKC signaling and correlated with enhanced sAPPα release [11,87]. Several studies have also demonstrated the participation and importance of the MAPK-ERK pathway in regulating the activity of α-secretase with the release of sAPPα blocked by the use of the ERK inhibitor, PD98059 [11,87]. In accord with those studies, we found that the levels of sAPPα are regulated in addition by ERK1/2 (Figure 8). Moreover, our results also implicate the involvement of PI3K in this process (Figure 9). Based on our results, we propose a model (Figure 10) summarizing the main findings of this study.

## 4. Materials and Methods

### 4.1. Materials

Most of the material used in this study was purchased as we reported earlier [19,24,46,88,89]. Phosphate Buffered Saline (PBS), nitrocellulose membranes, streptavidin-horseradish peroxidase (HRP) conjugate, Ponceau S solution, LY294002 hydrochloride, hydrogen peroxide solution, MISSION human ACHE (esiRNA1, EHU072891), recombinant human AChE (C1682, UniProt accession ID: C9JD78), recombinant human BDNF (B3795, UniProt accession ID: P23560), neostigmine methyl sulfate, PD98059, chelerythrine chloride, ACh, GI254023X were purchased from Sigma-Aldrich. PKI 14-22 amide was from R&D Systems. Sheep BDNF polyclonal antibody (PA1-18363), donkey anti-Sheep IgG (H+L) secondary antibody (HRP, A16041), amplex acetylcholine/acetlycholinesterase assay kit (A12217), goat anti-rabbit IgG (H+L) secondary antibody (HRP, 31466), mouse IgG isotype control, (mIgG), α-tubulin monoclonal antibody (DM1A), 3,3′,5,5′-tetramethylbenzidine (TMB), the Halt Protease and Phosphatase Inhibitor Cocktail, and lipofectamine 2000 transfection reagent were from ThermoFisher (Waltham, MA, USA). Goat anti-AChE antibody (ab31276), rabbit anti-Goat IgG H&L (HRP) (ab6741), and donkey anti-mouse IgG (HRP) (ab205724) were purchased from Abcam. Monoclonal Aβ antibody (sc-53822) and m-IgGκ BP-HRP, were from Santa Cruz Biotechnology (Dallas, TX, USA). Anti-Human Mouse sAPPα (2B3) IgG MoAb was purchased from IBL America (Minneapolis, MN, USA). The BCA protein assay kit, and the super signal west pico luminol (chemiluminescence) reagent were from ThermoFisher (Waltham, MA, USA). SignalSilence Control siRNA (Unconjugated, 6568) was purchased from Cell Signaling Technology (Danvers, MA, USA). Anti-Aβ (6E10, 1–16) antibody, anti-Aβ42 antibody that is reactive to the C-terminus of Aβ42, anti-Aβ40 antibody that is reactive to the C-terminus of Aβ40, and biotin anti-Aβ (6E10, 1–16) antibody were from BioLegend (San Diego, CA, USA).

### 4.2. Cell Culture

The human NSCLC cell lines, A549 (ATCC CCL-185) and H1299 (ATCC CRL-5803), were purchased from the American Type Culture Collection (ATCC, Manassas, VA, USA). Cells were seeded as we reported earlier [18,19,24,46,47,88,89] in 5 mL HyClone Dulbecco’s modified Eagle’s media/nutrient mixture F-12 (DMEM/F12) (GE Healthcare Life Sciences, Pittsburgh, PA, USA), supplemented with 10% Fetalgro bovine growth serum (FBS, RMBIO, Missoula, MT, USA), 50 U/mL penicillin, and 50 U/mL streptomycin (Invitrogen Life Technologies, Carlsbad, CA, USA) in 25 cm^2^ tissue culture flasks, and allowed to grow overnight in an incubator at 37 °C, 95% humidity, and 5% CO_2_. The cells were counted after trypan blue staining, with a hemocytometer.

When inhibitors were used, cells were treated with inhibitors against PI3K (LY294002, 14.5 μM), AChE (neostigmine methyl sulfate, 50 μM), ADAM10 (GI254023X, 10 μM), PKA (PKI 14-22 amide, 5 μM), PKC inhibitor (chelerythrine, 7.5 μM), MEK inhibitor (PD98059, 50 μM) as indicated.

### 4.3. ELISA

ELISAs were conducted as we reported previously [19,24,88,90]. Nunc MaxiSorp 96-well Flat Bottom plate (ThermoFisher) wells were coated with samples as indicated. The plates were incubated overnight at 4 °C on a shaker to allow binding to the plate wells. After the incubation, the wells were washed 4× with TBST, filled with 400 µL blocking buffer (110 mM KCl, 5 mM NaHCO_3_, 5 mM MgCl_2_, 1 mM EGTA, 0.1 mM CaCl_2_, 20 mM HEPES, 1% BSA, pH 7.4), and incubated overnight at 4 °C on a shaker. The wells were then washed 4× with TBST and 100 µL of sample at the desired concentration, was added to each well followed by incubation overnight at 4 °C on a shaker. TBST was then used to wash the wells 4× before proceeding in one of two ways: (1) biotinylated samples were analyzed by adding 100 µL streptavidin-HRP conjugate in TBST (1:2500 dilution) to the samples before incubating for 3 h at RT on a shaker, or (2) samples without biotin were analyzed by adding 100 µL TBST containing the primary antibody at the manufacturer’s recommendation and incubating for 3 h at RT on a shaker before washing 4× with TBST. The secondary antibody in 100 µL TBST was then added to the samples following the manufacturer’s recommendation and incubated for 1 h at RT on a shaker. Plates containing either biotinylated or non-biotinylated samples were then washed 5x with TBST followed by the addition of 100 µL TMB resulting in a blue color change. The reaction was stopped with 100 µL 2M H_2_SO_4_ after incubating at RT for 0.5–15 min, resulting in a yellow color change that was measured by absorbance at 450 nm. All absorbance measurements were in the linear range. To monitor non-specific binding, negative control wells on the plates included, for example, bound pure Aβ peptides [Aβ40-HFIP (AS-64128-05), Aβ42-HFIP (AS-64129-05)] or conditioned media, then adding all components, streptavidin-horseradish peroxidase and TMB, but without addition of biotin-6E10 antibodies. Some wells were coated with 2.5, 10, 50, 100, 500, and 5000 nM pure Aβ40 and Aβ42 peptides and probed with biotin-6E10 antibodies to allow conversion of the OD measurements to concentrations of bound material. Before analysis, the OD from the data was corrected for non-specific binding by subtracting the mean background absorbance for the negative controls. Typically, in control wells incubated on each plate, the background binding is about 10–15% of the maximum binding seen with addition of biotin-peptides or antibodies. Statistical analysis was determined by the GraphPad Prism 9.4.1 software. Data were expressed as the mean ± S.D. Three to five independent experiments were carried out in triplicate for each assay condition.

### 4.4. Quantitation of ACh Concentrations

The concentration of ACh was measured using the choline/acetylcholine assay kit (ab65345) according to the manufacturer’s recommendation. Briefly, media samples were added to wells followed by addition of the choline reaction mix in the absence or presence of AChE. The absorbance was then measured at 570 nm after incubation for 30 min at RT using a microplate reader. The amount of ACh was calculated by subtracting choline from total choline (choline + ACh).

### 4.5. Quantitative Determination of sAPPα

The concentration of sAPPα in the cell culture supernatant was measured using the quantitative sandwich enzyme immunoassay human sAPPα ELISA Kit (MyBioSource, San Diego, CA, USA, MBS915453) according to the manufacturer’s instructions. Briefly, plate wells have been pre-coated with an anti-sAPPα specific antibody. Following incubation of the wells with the samples, a biotin-conjugated antibody specific for sAPPα is then added. The signal was detected by incubation with avidin conjugated horseradish peroxidase (HRP) and a substrate solution.

### 4.6. Quantitation of Aβ

Aβ ELISAs were carried out according to previous protocols [91,92,93] and as we recently published [18] for determining the relative levels of Aβ. Briefly, Aβ1-40 and Aβ1-42 (Aβ40/42) were measured by two-site binding ELISAs using the anti-Aβ42 antibody that is reactive to the C-terminus of Aβ42, or anti-Aβ40 antibody that is reactive to the C-terminus of Aβ40, as the capture antibodies. After incubation with the media and washing the wells, biotinylated-anti-Aβ 6E10 (to Aβ1-16) antibody was added as the detection antibody and then the signal was quantitated using streptavidin-horseradish peroxidase and the TMB substrate. In addition, the same concentration of samples from the same treatments were added to ELISA wells and probed with only biotinylated-anti-Aβ 6E10 antibodies.

### 4.7. PKC Assay

The PKC activity was quantitated using the kinase activity assay kit (Abcam, Waltham, MA, USA, ab139437) In this solid phase ELISA, phosphorylation of a specific PKC synthetic peptide is detected using a polyclonal antibody.

### 4.8. PKA Assay

The PKA activity was measured using the solid-phase PKA activity assay kit (Invitrogen, Carlsbad, CA, USA, EIAPKA) that uses a PKA substrate immobilized to a microtiter plate. Two antibodies are then used to detect the substrate phosphorylated by PKA in the presence of ATP.

### 4.9. ERK Assay

ERK quantitation was measured using the ERK1/2 (pT202/Y204 + Total) ELISA kit (Abcam, ab176660) according to the instructions provided by the manufacturer. In brief, cell lysate samples were added to the wells followed by incubation with an antibody mix containing an affinity tag labeled capture antibody and a reporter conjugated detector antibody. The complex composed of the capture antibody/antigen/detector antibody is immobilized to the wells by an anti-tag antibody coated onto plate wells. After washing, the TMB substrate was added, and then the signal was detected by measuring the absorbance at 450 nm. Signals for phospho-ERK1/2 and total-ERK1/2 were normalized to cell number then the ratio of phospho-ERK1/2 to total-ERK1/2 for each treatment was determined and plotted.

### 4.10. PI3K Assay

Activated phosphorylated-PI3K p85 + total PI3K p85 in-cell ELISA kit (Abcam) was used according to the recommendations by the manufacturer as we recently reported [18,24]. Briefly, cells were cultured in 96-well plates then treated as indicated. Following treatment, the cells were fixed, and the wells were then incubated with a primary antibody targeting either total PI3K p85 (recognizes the total level of PI3K p85 proteins regardless of the phosphorylation state) or phosphorylated-PI3K p85 (recognizes p85 PI3K alpha/gamma phospho-tyrosine 467/199). Secondary HRP-conjugated antibodies were then added, and the signal was detected after addition of the developing solution. Crystal violet solution was then added to determine the relative number of cells in each well. Signals for phospho-PI3K and total-PI3K were normalized to cell number then the ratio of phospho-PI3K to total-PI3K for each treatment was determined and plotted.

### 4.11. Quantitation of mBDNF

Quantitation of mBDNF was carried out as we published previously [24] using the mBDNF rapid sandwich ELISA kit (Biosensis, Thebarton, Australia, BEK-2211-1P), that shows minimal cross-reactivity with proBDNF, according to the instructions provided by the manufacturer. The kit consists of a pre-coated mouse monoclonal anti-mBDNF capture antibody and a biotinylated anti-mBDNF detection antibody. The concentration of mBDNF in the media was calculated after addition of HRP-conjugated streptavidin and the TMB substrate.

### 4.12. Western Blotting

Samples of the media collected as indicated were analyzed according to our previous protocols [18,19,88]. Media samples were centrifuged and the supernatants were stored at −80 °C until further analysis. The protein concentrations were determined using the BCA protein assay kit. Following methods we reported previously [88], samples were boiled in 1× SDS, loaded and separated by SDS-PAGE on a 12% gel then transferred to a nitrocellulose membrane. The membrane was blocked in TBST buffer, pH 7.6, containing 5% nonfat milk for 6 h at 4 °C. The membrane was then incubated with the specific primary antibody in the blocking buffer, diluted as specified by the manufacturer at RT overnight with gentle shaking. After washing 3× with TBST, the membrane was incubated with a HRP linked secondary antibody in the blocking buffer, diluted according to the manufacturer’s recommendation. After washing 3× in TBST, the blots were developed using super signal west pico luminol (chemiluminescence) reagent and imaged with a Bio-Rad molecular imager.

### 4.13. SiRNA Transfection

Transfections were carried out according to our methods reported earlier [19,94]. The day before transfection, cells were seeded at a density of 2 × 10^4^ cells in 25 cm^2^ flasks. Control siRNA, AChE siRNA, were each mixed with Lipofectamine 2000 transfection reagent diluted in Opti-MEM Media (ThermoFisher) for 20 min at RT. The mixtures were then added to the cells at a final concentration of 100 nM for each siRNA and the cells were incubated at 37 °C for 12 h followed by the specific treatments as indicated. The cells were then allowed to incubate from 24 to 72 h at 37 °C. Cells exposed to Lipofectamine 2000 alone were used as a mock control. The media was used to quantitate ACh, mBDNF, Aβ40/42, sAPPα levels as described above. Each measurement represents the mean ± S.D. of three-five independent experiments, each performed in triplicate.

### 4.14. Statistical Analysis

The analysis was carried out as we previously reported [18,19,24,46,89]. Each experiment in this study was performed at least in triplicate and repeated a minimum of 3×. Statistical values are expressed as the mean ± Standard Deviation (SD). To evaluate the statistical differences, the Mann–Whitney or an ordinary one-way ANOVA followed by Tukey’s post-hoc multiple comparison test was performed. All the statistical tests were two-sided and a *p* value of <0.05 was considered statistically significant in all cases. GraphPad Prism (GraphPad Software, 9.4.1, Prism, La Jolla, CA, USA) was used for the statistical analysis.
